# Development and Usability of a Dashboard for Quality Monitoring and Resident-Centered Care in Australian Residential Long-Term Care: Mixed-Methods Study

**DOI:** 10.2196/80478

**Published:** 2026-04-29

**Authors:** Ronald Dendere, Gillian Stockwell-Smith, Michelle Lang, Murray Hargrave, Sara Mayfield, Leonard Charles Gray

**Affiliations:** 1 Centre for Health Services Research, Faculty of Health, Medicine & Behavioural Sciences, The University of Queensland, Level 5, Health Sciences Building, Herston Campus, Herston, Brisbane, Queensland, 4029, Australia, 61 7 3346 4636; 2 School of Nursing and Midwifery, Griffith University, Brisbane, Australia; 3 Regis Aged Care, Camberwell, Melbourne, Australia

**Keywords:** digital health, health IT, usability, care quality, quality indicator, health care dashboard, co-design, co-development, nursing home, residential aged care, long-term care, continuous quality improvement

## Abstract

**Background:**

The Australian National Aged Care Mandatory Quality Indicator Program (QI Program) requires government-subsidized residential aged care service providers to report quarterly data on a set of quality indicators. These indicators measure provider performance across specific domains of care and are intended to support continuous quality improvement. Health care dashboards can enhance the use of indicators by presenting data in interactive and intuitive formats that enable actionable insights.

**Objective:**

This mixed methods study aimed to develop an electronic dashboard to assist service providers’ use of QI Program data to measure, track, and improve the quality of resident care.

**Methods:**

A participatory design methodology was used to co-design and co-develop the dashboard. Initially, stakeholder participants for the co-design were identified. A combination of workshops, meetings, and email communications with co-design participants was then used to iteratively define and refine user requirements and to develop and improve the dashboard prototype. A 3-month pilot of the dashboard was conducted with a convenience sample of 30 end-users across 12 nursing homes and a post-pilot survey based on the System Usability Scale (SUS) was used to assess end-users’ perceptions of the dashboard usability.

**Results:**

The dashboard supports multiple user roles by enabling comparisons across homes and detailed views of all indicators for individual homes. A key feature is the ability to progressively view data at various levels of detail: groups of homes, individual homes, resident groups, and individual residents. The resident-level view enables more targeted, personalized care by helping staff identify and prioritize the specific indicators triggered by each resident. The average SUS score was 75.2 (SD 16.3), indicating good usability for the dashboard. Most survey respondents (12/14, 85.7%) were likely or extremely likely to recommend the dashboard to a colleague and agreed the dashboard would support the delivery of personalized care for residents. Almost all respondents (13/14) agreed or strongly agreed that the dashboard would assist with quality monitoring and improvement activities, and some pilot participants also made suggestions for incorporating the dashboard into those activities.

**Conclusions:**

This study demonstrates the potential value of a co-designed dashboard to support the use of quality indicator data in residential aged care. Limitations of the current prototype include short pilot duration, convenience sampling, and reliance on manual quarterly data uploads, which constrain generalizability and scalability. Future work should explore long-term integration of the dashboard into routine quality improvement processes and evaluate its impact on resident outcomes and care quality over time.

## Introduction

Quality indicators have become a common strategy in Australia and globally to support monitoring and improvement of quality in residential long-term care settings, including residential aged care facilities (also known as nursing homes) [1-6]. These metrics are quantitative measures for assessing care processes, structures, and outcomes to indicate how well care providers are performing in specific domains of care [7]. Indicators can facilitate the identification of care domains where quality is unacceptable or low and can enable tracking and comparison of quality at home/facility, organizational, regional, and national levels [1,7,8]. Indicators can also help identify top-performing services and enable the setting of evidence-based benchmarks to guide quality improvement efforts across long-term care settings [9].

Several countries use indicators for public reporting and comparing the performance of service providers [6]. In Australia, under the National Aged Care Mandatory Quality Indicator Program (QI Program), all government-subsidized service providers are required to report data on 14 indicators to the federal government every quarter of the financial year [10]. The program is designed to enable both the government and service providers to identify areas for improvement [10]. Five of the indicators also form a performance area used to determine a home’s overall rating in the public star rating system for residential aged care homes in Australia. Providers collect individual resident data in each home/facility using government-issued quality indicator spreadsheet templates that aggregate the data prior to submission. Typically, these data are sourced from different software, including clinical information systems, and risk and medication management software.

Quality indicators and their associated data must be understandable and actionable to fulfill their function as quality improvement tools [11,12]. Health information technologies (HITs), particularly dashboards, can be a key facilitator for this goal. Health care dashboards aggregate and analyze data about care recipients and care processes from multiple sources and present the data using graphics and other visuals to facilitate decision-making for health care professionals [13-15]. Dashboards are ubiquitous in acute care [13,16-18] but are increasingly utilized in the aged care sector in high-income countries, including Australia [19-21]. Although software products are available for collecting and reporting QI Program data, there is no published evidence of dashboards designed to support the practical use of these data for quality monitoring and continuous improvement by providers. Likewise, the scientific literature lacks evidence of dashboards developed specifically for QI Program indicators or demonstrating that providers are actively using these data to guide continuous quality improvement, as intended by the program. The QI Program’s quarterly collection of individual resident data presents an opportunity for providers to identify care domains requiring attention for each resident (ie, the indicators triggered) and to implement targeted care protocols accordingly. This resident-level approach not only has the potential to enhance outcomes for residents but also to improve overall provider performance within the QI Program. A dashboard would consolidate resident data into a single, accessible view and eliminate the need for providers to manually search across disparate software systems or navigate individual spreadsheets used for QI Program reporting.

Research shows that, like other HITs, involving end-users in the design and development of dashboards helps ensure the final product meets their specific needs. This collaborative approach also fosters a sense of ownership among end-users and other stakeholders, increasing the likelihood of adoption and long-term use [13,22-24].

The aim of this study was to develop and evaluate the usability of a dashboard to enable residential long-term care providers to use their mandated QI Program data to measure, monitor and improve the quality of resident care.

## Methods

### Study Design, Context, and Setting

This was a mixed methods study that used different methodologies for the dashboard design and development, and evaluation of usability. The dashboard described in this study is a component of a larger project that aimed to develop various standards-based health data, digital tools, and strategies for quality improvement [25]. The industry partner was Regis Aged Care (Regis), one of Australia’s largest aged care service providers [25].

### Ethical Considerations

Ethical approval was provided by the University of Queensland Human Research Ethics Committee (ID: 2022/HE002362). After accepting invitations to participate in the project, participants were supplied with an information sheet summarizing the project and a consent form for signing prior to data collection. Furthermore, researchers began each workshop/interview by explaining the project aims, procedures, and ethical considerations, including participants’ right to withdraw at any time. Participants were not compensated for participating in the project. All members of the research team adhered to institutional procedures governing research data storage, access, and privacy. Data were transcribed and deidentified using a commercial transcription service.

### Iterative Co-Design Approach

#### Overview

We used a participatory design methodology to develop the dashboard. The core principle of this user-centered approach is the direct involvement of people for whom a product is being designed in a collaborative co-design process [26]. This approach has been successfully used in the design of health care dashboards in general [27-29] and specifically, aged care dashboards [20,28,29].

**Figure 1.**
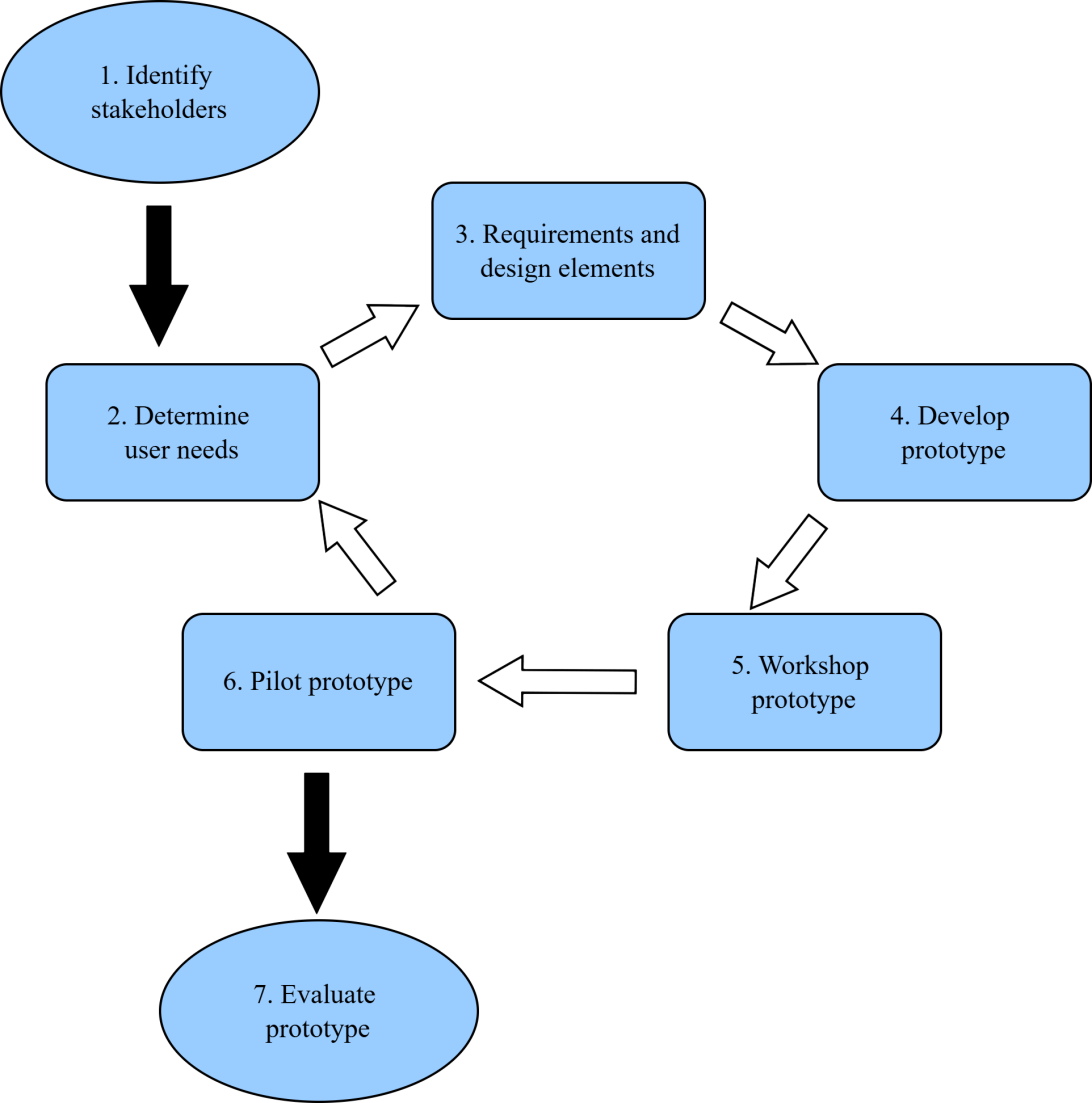
Co-design and evaluation stages.

Figure 1 summarizes the design, development, and evaluation process [27,28,30]. The process was largely iterative and entirely collaborative. Stages 1‐3 and 5 were conducted using a combination of in-person and online workshops to accommodate participants’ availability and geographical locations. Except for the first workshop, notes and outcomes of a prior workshop were reviewed at the beginning of each workshop to play these back, and confirm agreement of outcomes and decisions. We also leveraged fortnightly project committee meetings and email communications to expedite design decisions (all members of the project committee were participants in the co-design group).

#### Data Collection and Analysis

Workshop notes, transcripts of workshop recordings, and feedback received via email constituted the qualitative data for the project. As mentioned, the qualitative data were transcribed and deidentified using a commercial transcription service. Content analysis [31] was conducted using NVivo software [32] to derive the data sought at each stage (ie, identifying stakeholders, user needs, and requirements for the dashboard).

#### Identifying Stakeholders

To kickstart the project, we established a project committee consisting of the research team and 4 Regis staff members. The project committee collaboratively identified individuals and groups within Regis in executive and operational management roles for whom data aggregation and interrogation of the care, needs, and outcomes of residents formed a significant part of their responsibilities (ie, stakeholders). The Regis staff on the project committee then invited specific individuals from each group to participate in the co-design (ie, co-design participants).

#### Determining User Needs, Developing Requirements, and Design Elements for the Dashboard

A discussion of user needs was initiated in the first workshop by seeking out answers to three broad questions:

1. What is the purpose of the dashboard?
2. How will end-users use the dashboard?
3. What would end-users expect to achieve by using the dashboard?

Data collected during the first workshop were analyzed as described above (“Data Collection and Analysis” section) to compile a list of feature and functional requirements for the dashboard. The requirements were discussed and collaboratively refined during the second workshop. The final requirements list was translated into design elements. Design elements refer to user interface components (eg, buttons, icons, and menus), user experience patterns (eg, navigation patterns, workflows, and interaction models), layout of visuals, information architecture (organization of content) and system design (data structure and back-end logic that supports the front-end interactions/visuals).

#### Developing and Workshopping a Prototype

The first step in building the dashboard prototype was drawing up wireframes for each prospective view/page based on the design elements. A wireframe is a simple schematic created in the early stages of designing a digital product to help designers to communicate and co-design participants to visualize user-interfaces of digital products (eg, web pages, mobile apps, dashboards) [33,34]. We used a free, web-based diagramming tool to create the wireframes [35], which were distributed to participants via email to gather feedback and input. Design details requiring in-depth discussions were deferred for consideration during workshops to obtain consensus. This approach facilitated feedback and confirmed acceptability without requiring time-intensive coding.

Following consensus on the wireframes, development of the dashboard commenced by building the back end (ie, data model, logic, and calculations). The lead author developed the dashboard using Microsoft Power BI software (Microsoft Inc) [36], with technical and professional support from a credentialed Microsoft services provider [37]. Data for the dashboard were sourced from Regis’s quarterly QI Program data. Their national quality assurance manager, who was also a co-design participant, anonymized the residents using a unique code before supplying the data to the dashboard developer. The national quality assurance manager also supplied Regis’s IT team with the master spreadsheet containing the resident name and anonymization code pairs. Upon completion of the development, the dashboard file was sent to the Regis IT team who uploaded the master spreadsheet into the dashboard files prior to publishing. This approach preserved the privacy of residents while allowing individual residents to be linked between quarters and to be re-identified by Regis staff within the dashboard.

Following completion of the first prototype, and each update thereafter, we conducted online or in-person workshops to demonstrate the dashboard’s features and functions to the co-design group and gather feedback. During online workshops, the lead author guided participants through the dashboard workflow. During in-person workshops, participants were given hands-on access to the dashboard to explore and discuss its features and functionality. We employed the Think-Aloud method, an established approach for user interface evaluation in digital health [38], to gather real-time feedback. Participants were asked to verbalize their thoughts, observations, and opinions as they interacted with the dashboard. A member of the research team recorded these verbal responses and observations for subsequent analysis.

### Pilot and Usability Evaluation

To evaluate the dashboard usability, we conducted a pilot study designed to assess usability and perceived usefulness of the prototype, rather than to evaluate impact on care quality outcomes (which would require a longer evaluation period). Pilot participants were a convenience sample of 30 end-users in 12 nursing homes, consisting of general managers, regional general managers, clinical care managers, and clinical care specialists. Pilot participants were granted unrestricted access to the dashboard and encouraged to explore it freely. However, to facilitate structured engagement, we provided a monthly task list suggesting specific areas for exploration. Throughout the pilot, we met with participants during their routine monthly quality review meetings to monitor task progression and address user queries or technical issues.

At the conclusion of the pilot, we assessed the dashboard’s usability using the System Usability Scale (SUS) [39], a widely adopted, standardized instrument for evaluating perceived product usability, including HITs [40]. It comprises 10 items rated on a 5-point Likert scale, yielding an overall usability score between 0 and 100 [41,42]. SUS scores below 60 signal poor usability while scores in the 60‐69 range signal fair usability, 70‐79 signal good usability, 80‐84 signal excellent usability, and 85‐100 signal the best achievable usability [43]. In addition to the SUS items, the survey included questions assessing the perceived usefulness of the dashboard for person-centered care, its potential to support quality improvement, and suggestions for improvements. The survey was designed and administered using the Qualtrics platform.

## Results

### Co-Design and Iterative Approach

Overall, we conducted 5 workshops for Stages 1‐5: the first 2 workshops for Stages 1‐3 and the next 3 for Stages 2‐5 (see Figure 1). The combination of hands-on exploration and the Think-Aloud approach during in-person workshops was effective at stimulating discussion among the co-design group and facilitating development of new ideas for further enhancement of the dashboard.

#### Identifying Stakeholders

Overall, 9 stakeholders were identified across three broad categories reflecting the clinical care hierarchy of Regis:

1. Organization level: executive general managers, clinical governance and care committees, regional general managers, regional quality and improvement managers, regional clinical care specialist managers, quality and improvement teams, and clinical care specialists
2. Home level: general managers and clinical care managers
3. Resident level: registered nurses

Identifying stakeholders enabled the co-design group to consider their specific needs during the design of the dashboard. The co-design group consisted of the following roles (Textbox 1).

Textbox 1. **Roles of the co-design group.**

**Research**

- Informatics
- Health services and quality improvement
- Geriatric medicine
- Research nurse

**Regis aged care**

- Executive general manager
- Group manager
- Regional quality and improvement manager
- National quality assurance manager
- Regional clinical care specialist manager
- Quality and improvement manager

#### User Needs, Dashboard Requirements, and Design Elements

The final list of requirements is provided below.

1. The dashboard must display all current clinical and consumer experience indicators [10], that is, all except the “Workforce” indicator.
2. Home-level and resident-level end-users should have ability to select a specific home and period (ie, the “financial year” and “quarter”) whose indicators they intend to view.
3. Organization-level end-users should be able to select a group of homes and compare their performance for any indicator.
4. The dashboard must allow benchmarking against a national or organizational statistic (eg, average, median).
5. End-users should have the ability to drill-through the data and view details for each indicator per home. Drill-through is a term for functionality in dashboards that enables users to explore data at different levels of granularity by navigating from summary-level pages to detail-level pages. For example, navigating from an overview page showing all indicators for a specific home to a page showing details for the pressure injuries indicator (distribution of pressure injury stages, pressure injuries acquired outside the home, trends, etc).
6. The drill-through function must allow the viewing of individual residents, including the resident’s status for each indicator with clear indication of triggered indicators.
7. Access to resident-level data must be constrained by user credentials (eg, a registered nurse should only be able to view residents in the home in which they work).
8. The dashboard should have functionality to export visualizations/reports into PDF documents.

Design elements included drop-down lists for selecting variables to view (ie, homes, year, and quarter) and several combinations of “gauge,” “card,” and bar and line graph visuals in paginated views within the dashboard. Color was used extensively to convey key information visually—for example, to indicate a home’s performance relative to benchmarks. Co-design participants emphasized the importance of designing the dashboard to meet the diverse needs of end-users.

#### Developing and Workshopping a Prototype

**Figure 2.**
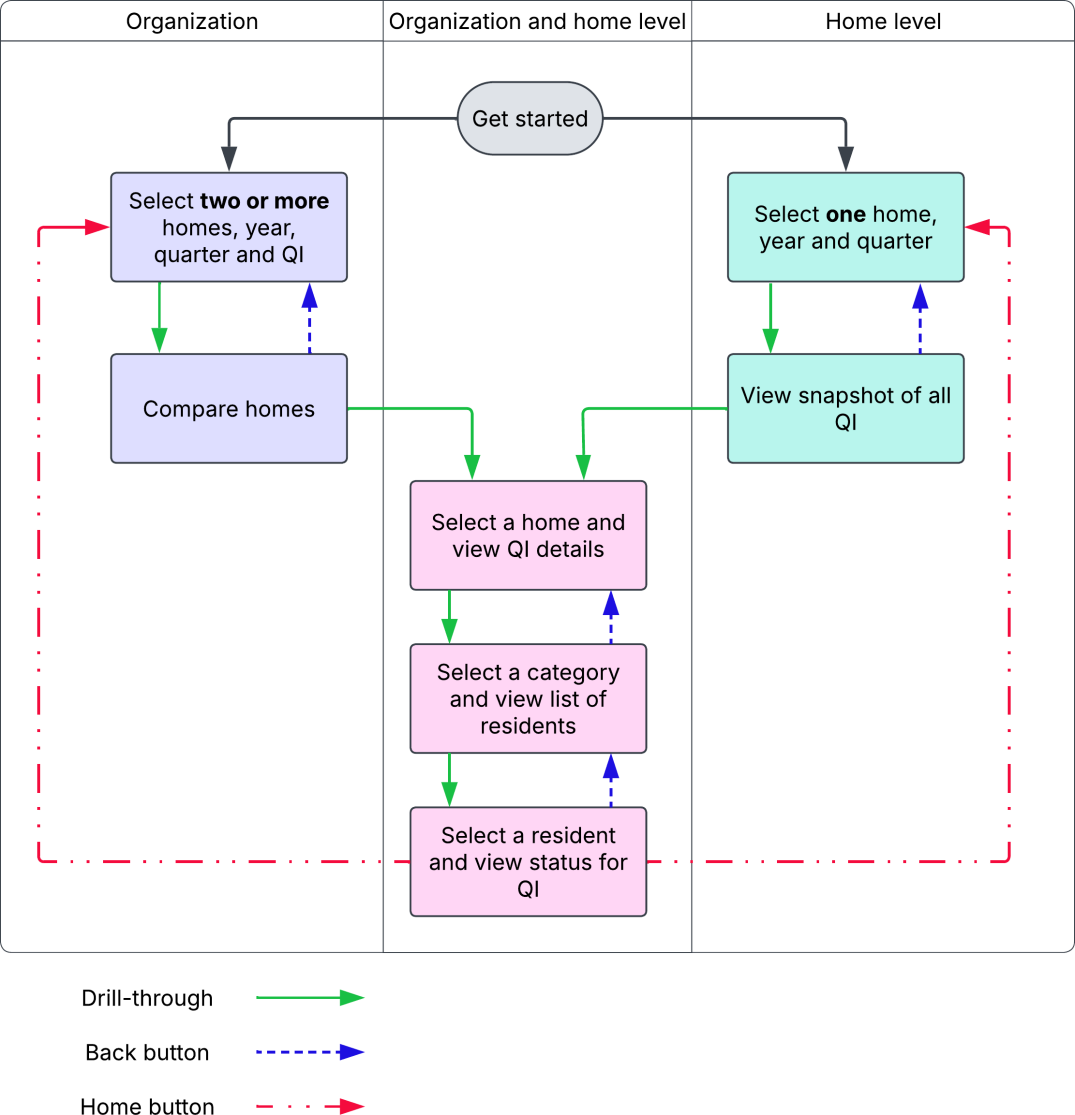
Organization-level and home-level workflows for the dashboard. QI: quality indicator.

**Figure 3.**
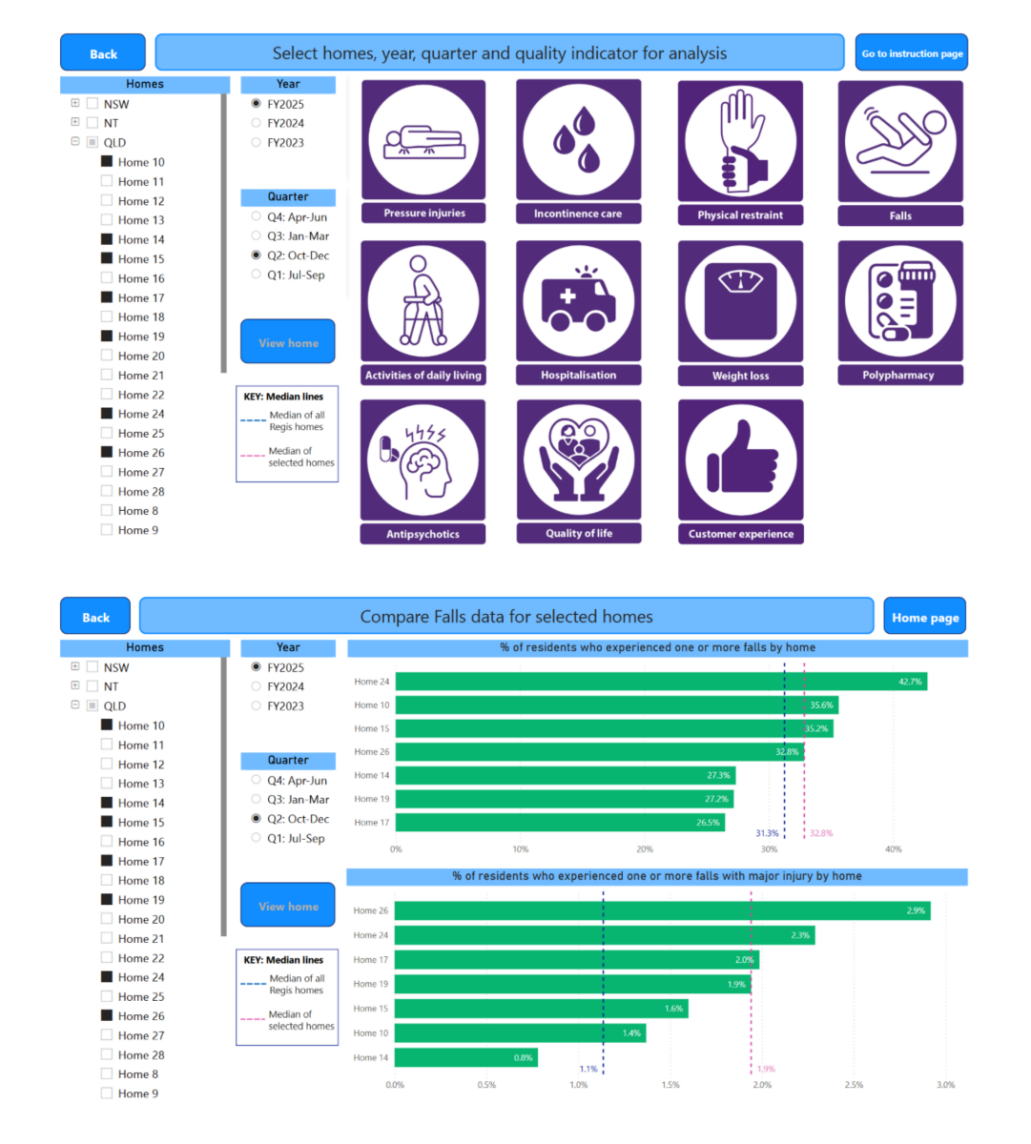
The (A) homepage for the organization-level version of the dashboard and (B) comparison of falls indicators. The names have been anonymized for illustrative purposes.

The final prototype incorporates 2 workflows (Figure 2) to fulfill the different needs of end-users: a common technique for tailoring health care dashboards for multiple use-cases using the same data [18,44]. The “organization-level” workflow, for users in the organization-level category (see the “Identifying Stakeholders” section in the Results), allows users to select several homes, the period, and a specific indicator for comparison. The “home-level” workflow, for users in the home- and resident-level categories, allows the selection and viewing of all indicators for a single home for a given period. Figure 3 shows the landing page for the organization-level workflow and an example of the subsequent comparison page when an indicator is selected.

**Figure 4.**
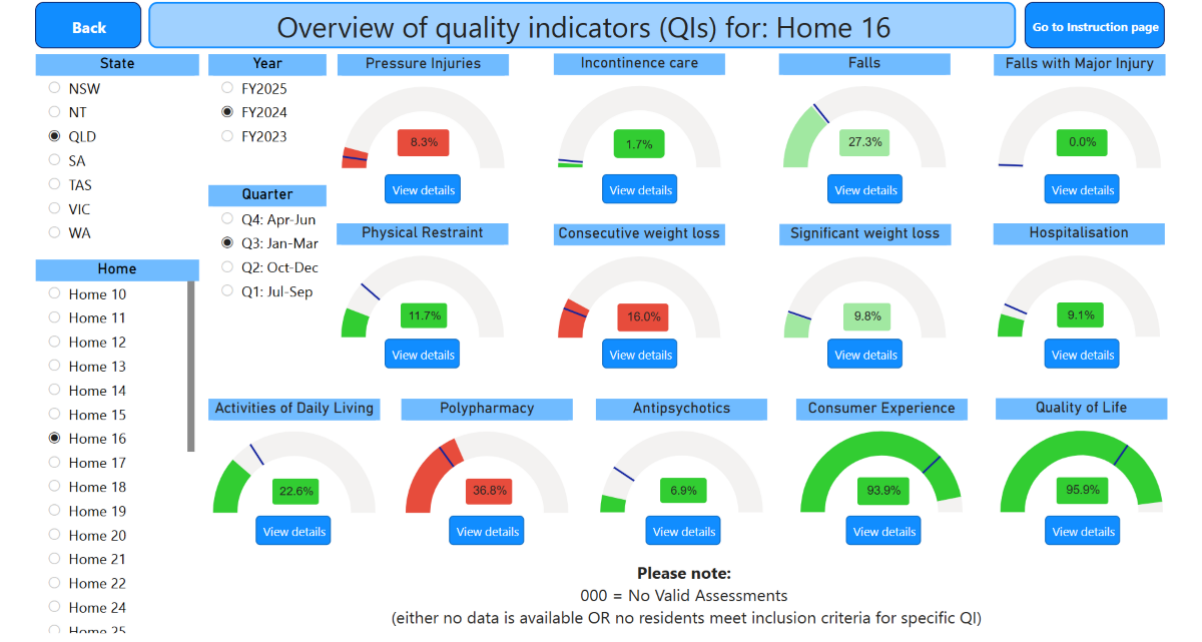
The homepage for the home-level version of the dashboard. The name of the selected home has been anonymized for illustrative purposes.

Figure 4 shows the landing page for the home-level workflow, which unlike the organization-level workflow, immediately displays an overview of all indicators after selection of a home and period. In this view, each home can be compared against a benchmark set for all homes in the organization. The benchmark was set as part of the co-design process to fulfill one of the requirements (see the “User Needs, Dashboard Requirements, and Design Elements” in the Results). It is indicated on the visual for each indicator by the dark line, and the actual value can be displayed by hovering the cursor over the line. The color scheme, also chosen during co-design, provides a visual indication of a home’s performance relative to the benchmark, with the intensity of color (ie, brighter/darker) indicating the percentage increase away from benchmark, as follows: (1) green signals performance above the benchmark, (2) amber signals performance equal to the benchmark, and (3) red signals performance below the benchmark.

**Figure 5.**
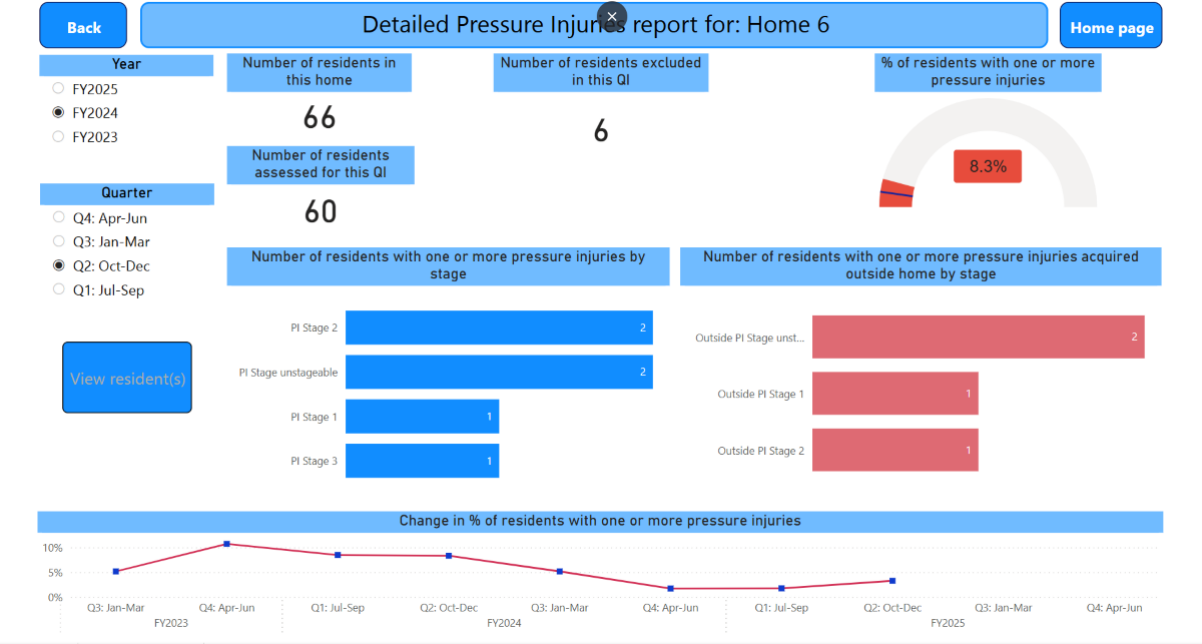
An example of a detailed QI (Pressure injuries) report for a specific home. The name of the home has been anonymized for illustrative purposes. QI: quality indicator.

**Figure 6.**
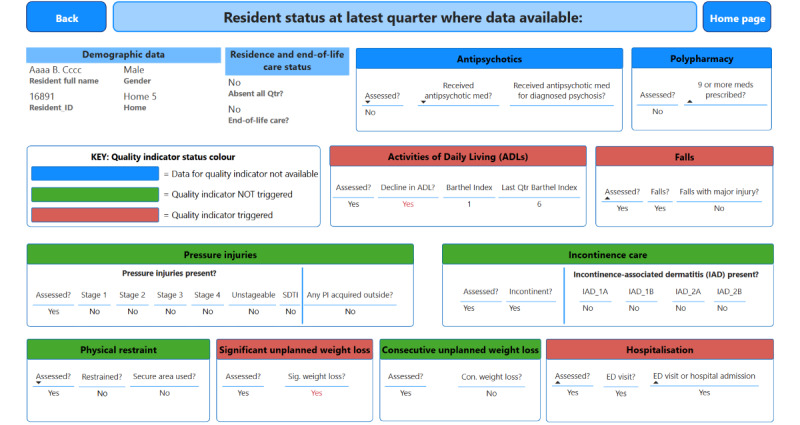
An individual resident’s QI status detail page. The resident’s name and home have been anonymized for illustrative purposes. QI: quality indicator.

Figure 5 shows an example of a drill-through to an indicator detail page. The indicator detail page displays all the reporting items required by the QI Program for that indicator. Figure 6 shows the resident-level data where a color scheme for each quality indicator title indicates the resident’s status as follows: (1) red signals that the resident has triggered the indicator (eg, the resident has a pressure injury), (2) green signals that the resident has not triggered the indicator (eg, the resident does not have a pressure injury), and (3) blue signals that the data for the indicator are not available (eg, the resident declined to be assessed for pressure injuries).

### Dashboard Pilot and Evaluation

Of the 30 individuals invited to participate in the pilot, 29 participants accessed the dashboard at least once during the pilot period, and 16 opened the usability survey (53% response rate) and 14 completed the survey (87.5% completion rate). The average SUS score was 75.2 (SD 16.3), indicating end-users thought usability of the dashboard was good. Among the completed surveys, 85.7% (12/14) of respondents reported being likely or extremely likely to recommend the dashboard to a colleague. An equal proportion (12/14) believed the dashboard would support the delivery of personalized care for residents. Additionally, 92.8% (13/14) agreed or strongly agreed that the dashboard would assist with quality monitoring and improvement activities within Regis.

The drill-through to individual resident-level views was well-received, with participants noting it could help staff quickly identify and prioritize relevant care domains, thereby promoting more person-centered care. In response to a survey item on integrating the dashboard into routine workflows, pilot participants suggested it could be useful for preparing internal quarterly reviews. They also recommended refreshing the data monthly to enable exporting dashboard views for inclusion in monthly clinical quality reports. Participants offered several suggestions for further improvements. However, some of these were contradictory, reflecting differences in personal preferences. For example, while some users preferred listing homes alphabetically when comparing performance, others favored sorting by descending indicator value to highlight the best- or worst-performing homes.

## Discussion

### Principal Findings

This article describes the design, development, and preliminary evaluation of a dashboard for quality indicator data. To our knowledge, it is the first published study to describe a dashboard specifically designed to support the Australian QI Program in aged care. Workshops were the primary collaboration method in the co-design process, complemented by a flexible, multi-channel approach that maintained momentum by enabling the prompt resolution of minor issues between scheduled sessions.

The multi-level drill-through capability is a key feature of the dashboard, which allows users to explore data at progressively finer levels of detail. This functionality supports a broad range of users across executive, managerial, and clinical roles. By providing secure access to resident-level data, the dashboard supports data-driven personalized care, which is associated with better resident outcomes and higher satisfaction and quality of life [45]. In this way, quality indicator data can be used as an operational asset for continuous quality improvement, enabling a bottom-up approach in which targeted care at the individual resident level may cumulatively contribute to improved performance at both home and organizational levels.

The dashboard requirements that were not specific to our use-case, that is, those that can be generalized to most health care dashboards, were consistent with those in the existing literature [14]. Like participants in other co-designed health care dashboards, co-design participants required functionality for selecting specific data subsets (homes or groups of homes, years, and calendar quarters) to view/compare indicators and multi-level drill-through capability, to have the ability to benchmark homes against group statistics, and to have secure access to resident-level data [14,19,46-48]. The design elements incorporated into the dashboard (radio buttons for navigation, color-coded visuals, line and bar charts, etc) were also consistent with existing literature [19]. The co-design and pilot participants found these design elements to be simple but powerful ways for conveying important details within the data as reported by users of other health care dashboards [19].

### Study Strengths and Limitations

A key strength of this study is the participatory, iterative development approach involving stakeholders across organizational executives, managers, and nurses. This enabled the tailoring the dashboard for real-world quality monitoring workflows and to support both performance oversight and resident-level care planning. Another strength is that the pilot was conducted in an operational aged care setting, providing an initial indication of feasibility and usability in practice.

This study has several limitations. First, the pilot evaluation was conducted with a convenience sample within a single aged care provider organization, and only 14 of the 30 participants completed the post-pilot survey. This limits generalizability and introduces the possibility of response and selection bias. Second, the 3-month pilot duration may be insufficient to understand long-term adoption, workflow integration, and sustained use. Third, the evaluation focused on perceived usability and usefulness but not effectiveness; we did not directly measure changes in care quality, staff decision-making, or resident outcomes attributable to dashboard use. Fourth, the prototype relied on manual data uploads and did not include automated data pipelines, which may limit scalability and real-world implementation readiness. Fifth, the dashboard operated on QI Program data, which is collected quarterly and may be too infrequent for timely monitoring of emerging issues in resident care.

### Implications for Practice and Future Development

Although the findings suggest potential for dashboards to make quality indicators more practical for monitoring care quality and supporting continuous improvement, broader implementation in practice will require technical and organizational development beyond this prototype stage. Future work should progress in 3 stages. First, implementation-ready infrastructure is needed, including a central data warehouse (or equivalent) and automated data pipelines from clinical information system sources. This would improve scalability, reduce manual workload, and enable stronger data quality controls (eg, completeness and timeliness checks). Second, dashboard functionality should be extended to support more frequent refreshes and enhanced analytics such as automated alerts and trend detection (improvements suggested by participants during this pilot). The divergent feedback on interface preferences during the pilot (eg, alphabetical vs performance-based sorting) suggests that usability in this context depends not only on simplicity, but also on flexibility. Future iterations should therefore include user-configurable interface features, such as selectable sort and display options, role-specific default views, and saved filters. These enhancements would better accommodate diverse workflows and user preferences across organizational, home-level, and clinical roles. Third, the dashboard should be evaluated in a larger, multi-site longitudinal study to measure not only usability and adoption but also the impact on quality improvement processes and resident outcomes. Organizations must establish robust governance strategies to mitigate the risk of “tunnel vision”—a phenomenon where attention becomes overly focused on the domains measured by implemented quality indicators at the expense of unmeasured aspects of care [49]. While this is a risk of using indicators for improvement more broadly [49], a dashboard with resident-level views may heighten the effect by directing clinical attention primarily toward the indicators presented on screen. Aged care organizations should also consider expanding the suite of dashboard quality indicators to complement the current QI Program and encompass a broader range of critical resident care domains.

### Specific Lessons

This study holds several lessons for informatics and the aged care sector. A multi-channel collaboration approach helped us gather feedback faster than relying solely on the workshops. The aged care industry, especially in Australia, is highly regulated, and providers occasionally have to attend to short-notice site visits and requests for information from regulators, necessitating immediate and intense activity by executive and operational managers. For this reason, it was common for some co-design participants to suddenly become unavailable for scheduled co-design workshops; however, the fortnightly meeting and emails provided additional avenues and opportunities to capture feedback and contributions. While fortnightly meetings may not be feasible for others, less frequent timeframes may be sufficient (eg, monthly or bimonthly). The feedback gathered from co-design participants using the Think-Aloud technique when interacting with the dashboard during in-person workshops provided richer data than online workshops. The Think-Aloud technique, which has been successful applied in HIT co-design in aged care [50], was particularly effective at stimulating insightful discussions and generation of ideas among co-design participants.

### Conclusion

In conclusion, dashboards co-designed with stakeholders have the potential to help aged care organizations operationalize quality indicator data for monitoring care quality and personalized care in residential aged care. Larger and longitudinal evaluations are required to determine their long-term impact on care quality and resident outcomes.

## Abbreviations

HIT: health information technology
QI Program: National Aged Care Mandatory Quality Indicator Program
SUS: system usability scale

## References

[1] Frijters DHM, van der Roest HG, Carpenter IGI (2013). The calculation of quality indicators for long term care facilities in 8 countries (SHELTER project). BMC Health Serv Res.

[2] Loureiro N, Martin JI (2024). Quality indicators for residential long-term care for the elderly: a scoping review. RASP.

[3] Igarashi A, Eltaybani S, Takaoka M, Noguchi-Watanabe M, Yamamoto-Mitani N (2020). Quality assurance in long-term care and development of quality indicators in Japan. Gerontol Geriatr Med.

[4] Inacio MC, Eshetie TC, Caughey GE (2023). Quality and safety in residential aged care: an evaluation of a national quality indicator programme. Intern Med J.

[5] Inacio MC, Lang C, Caughey GE (2020). The Registry of Senior Australians outcome monitoring system: quality and safety indicators for residential aged care. Int J Qual Health Care.

[6] Osińska M, Favez L, Zúñiga F (2022). Evidence for publicly reported quality indicators in residential long-term care: a systematic review. BMC Health Serv Res.

[7] Mainz J (2003). Defining and classifying clinical indicators for quality improvement. Int J Qual Health Care.

[8] Zimmerman DR (2003). Improving nursing home quality of care through outcomes data: the MDS quality indicators. Int J Geriatr Psychiatry.

[9] Schwabe J, Caughey GE, Jorissen R (2024). Setting standards in residential aged care: identifying achievable benchmarks of care for long-term aged care services. Int J Qual Health Care.

[10] (2024). About the QI Program. Australian Government Department of Health, Disability and Ageing.

[11] Barbazza E, Klazinga NS, Kringos DS (2021). Exploring the actionability of healthcare performance indicators for quality of care: a qualitative analysis of the literature, expert opinion and user experience. BMJ Qual Saf.

[12] Berwick DM, James B, Coye MJ (2003). Connections between quality measurement and improvement. Med Care.

[13] Helminski D, Sussman JB, Pfeiffer PN (2024). Development, implementation, and evaluation methods for dashboards in health care: scoping review. JMIR Med Inform.

[14] Rabiei R, Almasi S (2022). Requirements and challenges of hospital dashboards: a systematic literature review. BMC Med Inform Decis Mak.

[15] Stadler JG, Donlon K, Siewert JD, Franken T, Lewis NE (2016). Improving the efficiency and ease of healthcare analysis through use of data visualization dashboards. Big Data.

[16] Buttigieg SC, Pace A, Rathert C (2017). Hospital performance dashboards: a literature review. J Health Organ Manag.

[17] Dowding D, Randell R, Gardner P (2015). Dashboards for improving patient care: review of the literature. Int J Med Inform.

[18] Young L, Vogelsmeier A (2024). Quality dashboards in hospital settings: a systematic review with implications for nurses. J Nurs Care Qual.

[19] Siette J, Dodds L, Sharifi F (2023). Usability and acceptability of clinical dashboards in aged care: systematic review. JMIR Aging.

[20] Silva SSM, Wabe N, Nguyen AD (2025). Development of a predictive dashboard with prescriptive decision support for falls prevention in residential aged care: user-centered design approach. JMIR Aging.

[21] Ludlow K, Westbrook J, Jorgensen M (2021). Co-designing a dashboard of predictive analytics and decision support to drive care quality and client outcomes in aged care: a mixed-method study protocol. BMJ Open.

[22] Murphy DR, Savoy A, Satterly T, Sittig DF, Singh H (2021). Dashboards for visual display of patient safety data: a systematic review. BMJ Health Care Inform.

[23] van Elten HJ, Sülz S, van Raaij EM, Wehrens R (2022). Big data health care innovations: performance dashboarding as a process of collective sensemaking. J Med Internet Res.

[24] van de Baan FC, Lambregts S, Bergman E, Most J, Westra D (2023). Involving health professionals in the development of quality and safety dashboards: qualitative study. J Med Internet Res.

[25] (2023). Aged Care Data Compare to put quality benchmarking to the test. Digital Health Cooperative Research Centre.

[26] Simonsen J, Robertson T (2013). Routledge International Handbook of Participatory Design.

[27] Ratwani RM, Fong A (2015). “Connecting the dots”: leveraging visual analytics to make sense of patient safety event reports. J Am Med Inform Assoc.

[28] Burningham Z, Lagha RR, Duford-Hutchinson B (2022). Developing the VA Geriatric Scholars Programs’ clinical dashboards using the PDSA framework for quality improvement. Appl Clin Inform.

[29] Esquer Rochin MA, Gutierrez-Garcia JO, Rosales JH, Rodriguez LF (2021). Design and evaluation of a dashboard to support the comprehension of the progression of patients with dementia in day centers. Int J Med Inform.

[30] Woods L, Cummings E, Duff J, Walker K (2018). Conceptual design and iterative development of a mHealth app by clinicians, patients and their families. Stud Health Technol Inform.

[31] Elo S, Kyngäs H (2008). The qualitative content analysis process. J Adv Nurs.

[32] NVivo. Lumivero.

[33] Guilizzoni P (2025). What is a wireframe? A guide for non-designers. Balsamiq.

[34] What is a wireframe? + How to create one. Cousera.

[35] About draw.io. Draw.io.

[36] Power BI. Microsoft.

[37] Agile Insights.

[38] Jaspers MWM, Steen T, van den Bos C, Geenen M (2004). The think aloud method: a guide to user interface design. Int J Med Inform.

[39] Brooke J (1996). Usability Evaluation in Industry.

[40] Almasi S, Bahaadinbeigy K, Ahmadi H, Sohrabei S, Rabiei R (2023). Usability evaluation of dashboards: a systematic literature review of tools. Biomed Res Int.

[41] Lewis JR, Sauro J The factor structure of the System Usability Scale.

[42] Bangor A, Kortum PT, Miller JT (2008). An empirical evaluation of the System Usability Scale. Int J Hum Comput Interact.

[43] Bangor A, Kortum P, Miller J (2009). Determining what individual SUS scores mean: adding an adjective rating scale. J Usability Stud.

[44] Mlaver E, Schnipper JL, Boxer RB (2017). User-centered collaborative design and development of an inpatient safety dashboard. Jt Comm J Qual Patient Saf.

[45] Millar RJ, Diehl C, Blake E, Fakeye O, Kusmaul N (2024). Nursing home characteristics and resident quality of care outcomes: a scoping review. JLTC.

[46] Ghazisaeidi M, Safdari R, Torabi M, Mirzaee M, Farzi J, Goodini A (2015). Development of performance dashboards in healthcare sector: key practical issues. Acta Inform Med.

[47] Randell R, Alvarado N, McVey L (2019). Requirements for a quality dashboard: lessons from national clinical audits. AMIA Annu Symp Proc.

[48] van Deen WK, Cho ES, Pustolski K (2019). Involving end-users in the design of an audit and feedback intervention in the emergency department setting - a mixed methods study. BMC Health Serv Res.

[49] Lester HE, Hannon KL, Campbell SM (2011). Identifying unintended consequences of quality indicators: a qualitative study. BMJ Qual Saf.

[50] Cole AC, Adapa K, Khasawneh A, Richardson DR, Mazur L (2022). Codesign approaches involving older adults in the development of electronic healthcare tools: a systematic review. BMJ Open.

